# Three Dimensional Tracking of Exploratory Behavior of Barnacle Cyprids Using Stereoscopy

**DOI:** 10.1007/s13758-012-0050-x

**Published:** 2012-08-21

**Authors:** S. Maleschlijski, G. H. Sendra, A. Di Fino, L. Leal-Taixé, I. Thome, A. Terfort, N. Aldred, M. Grunze, A. S. Clare, B. Rosenhahn, A. Rosenhahn

**Affiliations:** 1 Institute of Functional Interfaces, KIT, P.O. Box 3640, 76021 Karlsruhe, Germany; 2 Applied Physical Chemistry, University of Heidelberg, INF 253, 69120 Heidelberg, Germany; 3 School of Marine Science and Technology, Newcastle University, Newcastle upon Tyne, NE1 7RU UK; 4 Institute for Information Processing, Leibniz University Hannover, Appelstr. 9A, 30167 Hannover, Germany; 5 Institute of Inorganic and Analytical Chemistry, Goethe University Frankfurt, 60438 Frankfurt, Germany

## Abstract

Surface exploration is a key step in the colonization of surfaces by sessile marine biofoulers. As many biofouling organisms can delay settlement until a suitable surface is encountered, colonization can comprise surface exploration and intermittent swimming. As such, the process is best followed in three dimensions. Here we present a low-cost transportable stereoscopic system consisting of two consumer camcorders. We apply this novel apparatus to behavioral analysis of barnacle larvae (≈800 μm length) during surface exploration and extract and analyze the three-dimensional patterns of movement. The resolution of the system and the accuracy of position determination are characterized. As a first practical result, three-dimensional swimming trajectories of the cypris larva of the barnacle *Semibalanus balanoides* are recorded in the vicinity of a glass surface and close to PEG2000-OH and C_11_NMe_3_^+^Cl^−^ terminated self-assembled monolayers. Although less frequently used in biofouling experiments due to its short reproductive season, the selected model species [Marechal and Hellio (2011), Int Biodeterior Biodegrad, 65(1):92–101] has been used following a number of recent investigations on the settlement behavior on chemically different surfaces [Aldred et al. (2011), ACS Appl Mater Interfaces, 3(6):2085–2091]. Experiments were scheduled to match the availability of cyprids off the north east coast of England so that natural material could be used. In order to demonstrate the biological applicability of the system, analysis of parameters such as swimming direction, swimming velocity and swimming angle are performed.

**Electronic supplementary material:**

The online version of this article (doi:10.1007/s13758-012-0050-x) contains supplementary material, which is available to authorized users.

## Introduction

Since 2008, the most successful of the heavy-metal based antifoulants, tributyltin (TBT) [3], has been banned and research has since focused on alternative strategies to mitigate the undesired accumulation of biomass on vessels submerged in the marine environment. In order to develop environmentally benign coatings, a better understanding of the colonization mechanisms of the target organisms is required. Ideally, improved knowledge of surface cues that promote settlement of fouling species as well as investigation of surface cues that repel settlement will lead towards effective but environmentally inert antifouling materials. For most biofoulers it is not the macroscopically visible adult organism that is relevant in this context, but the colonization stage, which is responsible for initial surface attachment [4].

Barnacles, as one of the most prevalent marine fouling groups [5], contribute significantly to increased hydrodynamic drag and higher fuel consumption of vessels. Much of the research on settlement behavior of thoracican barnacles has been driven by the perceived need for a better understanding of larval settlement in order to interfere with the process.

In barnacles, the cyprid, or cypris larva is the colonization stage. The evolution of barnacles for a sessile mode of life is best epitomized at this stage. The cyprid, armed with a complex array of sensory setae [6], has a remarkable ability to search the substratum using reversible adhesion to locate a suitable place to settle for the remainder of its life [5]. Two-dimensional tracking has already been used to derive statistically relevant data from the behavior of cyprids on surfaces, revealing differences in exploratory behavior depending on surface chemistry [7]. However, the three dimensional nature of cyprid behavior makes gathering information of satisfactory quality a challenging task. Particularly, distinguishing between cyprids that are actively swimming, passively floating, or exploring surfaces is next to impossible on the basis of 2D data alone. Thus, 3D tracking techniques are a natural and necessary progression.

In the case of small microorganisms such as bacteria or algal spores, digital holographic microscopy has been used to extract 3D traces of moving objects [8–11]. It has also been successfully implemented to reveal predator–prey behavior in dinoflagellates [12]. Holography, however, requires significant expertise and infrastructure if it is to be successfully applied. Alternatively, stereoscopy is a technique able to track objects as diverse as people [13, 14], small particles in particle tracking velocimetry [15] or dusty plasmas under microgravity [16]. Compared to holography, a major advantage of stereoscopy is that no reconstruction of the recorded data is required.

We developed a system with two consumer camcorders which allows the determination of three dimensional trajectories of barnacle cyprids based on stereoscopy. The hardware setup, as well as the technical issues for obtaining the 3D swimming trajectories, are described in this article, including an empirical error analysis. We tested the system with cyprids of *Semibalanus balanoides* on chemically different surfaces (glass, PEG2000-OH and C_11_NMe_3_^+^Cl^−^) and first trajectories are visually presented. Several descriptive features are extracted from the trajectories in order to show the feasibility of the system in biological applications.

## Materials and Methods

### Theoretical Framework for the Epipolar Geometry

**Fig. 1:**
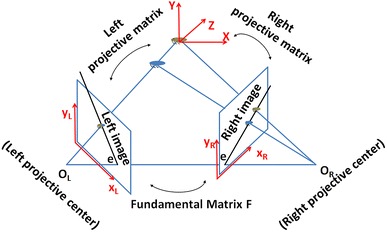
A general geometrical model of a stereoscopic system, showing the main idea of stereoscopy and the common *y* axis (*red*) to all the coordinate systems of our setup

The idea of using stereoscopy (Fig. 1) to determine the three dimensional (3D) position of a microorganism is based on the observation of its two dimensional (2D) position in the two cameras and the combination of the information from both images [17]. The relationship between the 2D detected points in the camera (**x**) and their real 3D positions (**X**) is given by the projection matrix **P** through the expression.

1where **x** = *(x, y, s)* represents the image point in homogeneous coordinates, meaning (*x/s, y/s*) in Euclidean space, and **X** = (*X, Y, Z,* 1) are the homogeneous coordinates of the position with real coordinates (*X, Y, Z*) [17]. The projection matrix is defined as2where *k*_*x*_ and *k*_*y*_ are the pixels per unit distance in pixel coordinates, *f* is the focal length and (*x*_0_, *y*_0_) is the principal point. The elements of the matrix **R** contain all the information about the rotation and translation of the camera with respect to any coordinate system, and are called rotation-translation matrix or matrix of extrinsic parameters.

Theoretically, a set of at least six 3D points **X**_*n*_ and their projection on the image plane **x**_*n*_ are necessary to calculate the projection matrix **P**, which can be computed as a direct linear transformation by optimizing the equivalent linear system given by3

With the projection matrix **P** it is possible to backproject any 2D point to a 3D projection ray. If this is done on both cameras, the intersection of the corresponding projection rays will represent the reconstructed 3D position.

In Pluecker coordinates [18] each 3D line is represented by two vectors, namely L: (**d**, **m**), where the first one (**d**) is a normalized 3D vector in the direction of the line (with ||**d**|| = 1) and the second one (**m**), called moment, is a perpendicular vector defined as **m** = **X** × **d**, with **X** being the position of any point on the line expressed as a 3D vector. This notation presents certain advantages. Considering two lines L_1_: (**d**_1_, **m**_1_) and L_2_: (**d**_2_, **m**_2_), the distance between any point **X** and the line L can be computed as4

Therefore, the point on *L*_1_ which is closest to *L*_2_ can be calculated as5

When there is an intersection of the two lines *L*_1_ and *L*_2_, then6

Two corresponding projection rays usually do not intersect due to errors in the calibration, the detection of the particle, or the quantization. In this case, the closest point between both skewed lines *L*_1_ and *L*_2_ can be calculated as7

Assuming a good calibration, the 3D spatial resolution of the system can be estimated by considering a deviation in the components of vector **x** and its homologous **x’** (the other camera), which can be mathematically expressed in Euclidean space as8

If the values Δ*x* and Δ*y* are added to both vectors **x** and **x′** and the 3D points are calculated for all combinations, the bounded 3D region defines the spatial resolution. However, this region is not spatially uniform for all values of **x** and **x′** and the extreme values should be considered to determine the minimum, maximum and mean resolution.

### Stereoscopy Setup

**Fig. 2:**
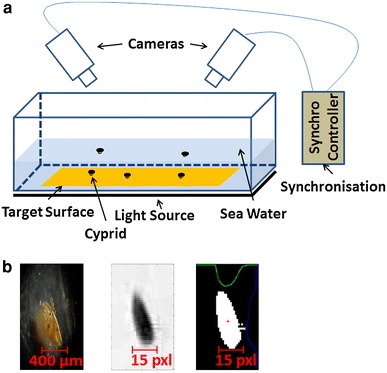
**a** Setup of the system used to carry out the experiments and **b** the target organism [34], its projection in the video, and its binary image

The test setup consists of two cameras (Sony HDR-XR550, square pixels, with resolution 1440 × 1080 @ 25 fps, focal distance 3.8–38 mm) connected with a synchronization device (ste-fra LANC V3.0 M, digidat). Both cameras were manually focused on the sample using telephoto mode, with a sample-camera distance of approximately 0.8 m. The test surface is placed in a rectangular cell culture vessel (quadriPERM^®^, Sigma-Aldrich) filled with artificial seawater (Instant Ocean^®^) and the target organisms. The volume of interest was illuminated from below using a light source (3.5″ × 6″ LED area backlight, Metaphase Tech). A schematic representation of the setup is presented in Fig. 2a.

The video files recorded during the experiments were split into single frames, and their color format (24 bit RGB) was then converted to grayscale (8 bit). A “mean” background image was calculated by averaging five frames from the beginning, five frames from the middle and five frames towards the end of the video stream, in which the moving objects were present and thus filtered out. This frame, containing only the “static” parts of the image, was subtracted from every frame which was analyzed, thus eliminating most of the artifacts from container borders, edges and corners. To avoid a potential elimination of attached cyprids, the videos were manually inspected beforehand. In order to simplify and accelerate further calculations and processing, each image was transformed from grayscale to binary using the thresholding method described by Otsu [19]. The centroid of the object of interest in each image was estimated applying an image moment calculation.

Since a low density of cyprids was used for these experiments we applied single object tracking to generate the swimming trajectories. An automatic algorithm was used where the user selected the starting position of the cyprid and its positions in the subsequent frames were determined evaluating the minimum Euclidean distance to all the candidate cyprids. The process was supervised by the user to avoid erroneous detection in case of crossing or overlapping trajectories. For the future, efficient multi object tracking approaches will be applied as described in [20].

### System Calibration

In order to determine the projection and fundamental matrices for a given experimental situation, a calibration needs to be performed prior to the tracking experiment. During the calibration an object, with marks or points, the positions of which are known in real-world coordinates, is used to determine the corresponding positions in image coordinates for both camera images (left and right frame). The calibration object needs to possess at least 6 known points (in our case we used 8) in order to provide enough information to solve the system of Eq. (3). During the calibration, these points (**X**_**real**_) are selected from different positions of the calibration object. The corresponding positions in image coordinates are detected in the left (**x**_**left**_) and the right frames (**x**_**right**_). Using the correspondences, the projection matrixes for the left (**P**_**left**_) and the right (**P**_**right**_) camera are calculated by solving Eq. (3).

**Fig. 3:**
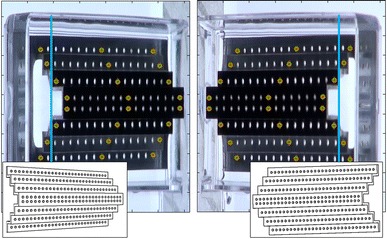
Left and right camera images of the applied calibration object. Several matched points which can be used to calculate the camera models are shown in *green*. In our experiment, because of the limited depth of the container, only underwater points are considered (*cyan line* indicating the water line), in order to compensate the difference in the refractive indexes (air, water, etc.) [35]

For the present work we use a 3D CAD software designed calibration object with 128 points (back-illuminated holes with a diameter of 1 mm) equidistantly distributed across four planes (Fig. 3) which was fabricated using a computer programmed CNC machine. The calibration object has the same x and y dimensions as the objective slides (26 mm × 76 mm) and can easily be exchanged against a coated objective slide after calibration is finished.

### Cyprids and Cyprid Collection

The tests described in this work were performed in the School of Marine Science and Technology of Newcastle University, UK. As target organisms, cyprids from the species *Semibalanus balanoides* were used. Cyprids were collected by plankton tow from the harbor wall at the Dove Marine Laboratory, Cullercoats, NE England and transported to the University where they were transferred to filter-sterilized (0.22 μm) seawater and stored at 6 °C in glass beakers. Cyprids sized ca. 800 μm, which do not feed, were used within 2 weeks of collection. The experiments were performed at room temperature of around 22 °C, and cyprids were left undisturbed for 10 min prior to each measurement in order for them to adapt to the change of water temperature.

### Surface Preparation

Regular glass slides as well as self-assembled monolayers (SAMs) on gold-coated glass slides were applied as test surfaces. The employed chemistries on the SAMs, described in Table 1, were used along with Nexterion^®^ glass (Schott) as frequently used standard [21, 29].

**Table 1:** Surfaces used for the experiment: PEG, Glass and TMA

Chemistry	Name	Water contact angle (°)	Ellipsometric thickness (nm)	Short name
HS–(CH_2_)_2_–(O–(CH_2_)_2_)_44_–OH	Hydroxy-PEG 2000-thiol	27	7.38	PEG
Acid washed glass	Nexterion® glass (Schott)	15	–	Glass
HS–(CH_2_)_11_–NMe_3_^+^Cl^−^	11-Mercaptoundecyl-1-trimethylammonium chloride	34	1.71	TMA

The surfaces were selected because of their different attractiveness for barnacle cyprids. The corresponding formulas are shown, as well as their thickness determined by spectral ellipsometry. Static water contact angles are also shown

## Results and Discussion

### Characterization of the Accuracy in the Position Determination

During calibration it became obvious that the estimation of the exact coordinates of the calibration points at sub-pixel level is difficult and can lead to calibration errors. In addition, illumination and contrast between the swimming object and the background affect the exact estimation of the object’s coordinates. Depending on the reflectivity of the surface, mirror aberrations might also occur, which combined with the morphing of the non-spherical objects of interest (Fig. 2b) can induce inaccuracies in the detected position. Several tests (*see Online Resource 1*) were performed in order to characterize the system’s behavior when an error is introduced. By this analysis, the relationship between the error in the real position (in mm) and in the position of the object in the left and the right frames (in pixel) was empirically determined.

**Fig. 4:**
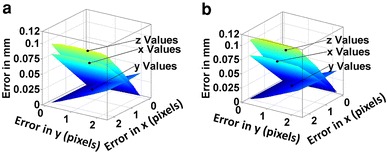
Influence of error in position determination in the image frames on the resulting error in real life coordinates of the object position in mm, when an error is introduced in the*left* (**a**) or*right* (**b**) frame. The three planes resemble the 3D position error in the X, Y and Z position, respectively. The maximum error in image coordinates presented in this figure is 2 pixels. For higher values (up to 14 pixels) please consult *Online Resource 1*, Fig. 2

A worst-case scenario in the centroid calculation was simulated by artificially introducing a worst-case-error of 14 pixels in the center of mass estimation, thus lying completely outside of the cyprid body (Fig. 2). This inaccuracy in the position in image coordinates introduced an error in the position in real coordinates of 0.5 mm (≈50 % of physical cyprid size) in *X* and *Z,* and 0.2 mm (≈20 % of physical cyprid size) in *Y* (*see Online Resource 1,* Fig. 2). Based on our observations, this worst-case error never occurred in the experiments and an average error of 0.1 pixels was typical for this application and the used configuration of the setup. This means that the average error of the system related to the size of the cyprids (Fig. 4) is around 0.004 mm (≈0.4 % of physical size of *Semibalanus balanoides* cyprids). If we consider this average error in the 3D position determination of the points as Δp, then the error in the distance between two consecutive trajectory points will be Δd = 2Δp = 0.008 mm. Then the average error in the velocity becomes Δv = 2Δp/T = 0.2 mm/s, where T is the sampling period of the images (T = 1/25 s). This accuracy is sufficient for the desired experiments to record and statistically analyze 3D trajectories of barnacle cyprids.

**Fig. 5:**
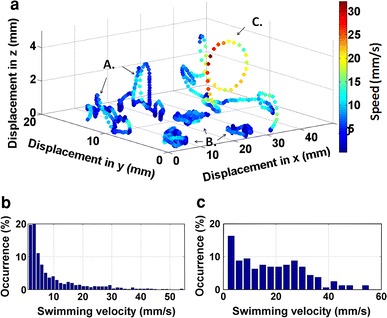
Example trajectories of three distinct motion patterns (A., B. and C.) of *Semibalanus balanoides* cyprids, exploring a glass surface (**a**). The*color coding* represents the cyprid velocity at a given position according to the color bar (*red* meaning higher, *blue*—slower velocity). Histogram of the accumulated swimming velocity obtained from all trajectories (**b**) and only from parts of trajectories corresponding to active swimming (**c**)

In the following, we demonstrate the applicability of the system by tracking the swimming behaviour of cyprids (*Semibalanus balanoides*). Figure 5a shows some typical 3D trajectories, each with a total duration of 10 s. The color of each position indicates the swimming velocity of the cyprids at each point as given by the color bar. Figure 5b shows the swimming velocity histogram of all the traces obtained from the experiments. In Fig. 5c, the velocities only of actively swimming cyprids are shown (swimming speeds lower than 1 mm/s have been omitted to restrict the histogram to real motions rather than small cyprid body movements). From these two histograms it can be seen that the maximum speed in these particular measurements is ca. 55 mm/s and the mean swimming speed (of actively swimming cyprids) is ca. 20–25 mm/s. However, the distribution is very broad, indicating that cyprids swim at different speeds rather than a preferred velocity. These obtained values are in good agreement with previous reports by other groups [22–25].

In Fig. 5a different motion patterns can be detected, which we labeled as A, B and C. Cyprids swimming in a region far away from the surface (2–5 mm distance) show relatively high velocities and rather directed motions. From the color coding it can be seen that the trajectory labeled “C” exhibits velocities of up to 15–30 mm/s. Traces indicated by “A” have slightly lower speeds in the range 5–15 mm/s. These values are below the mean velocity calculated (Fig. 5c).

When the cyprids are active in the vicinity (<1 mm distance) of the surface, the swimming velocities are also low (5–7 mm/s). The “B” traces have very limited spatial displacements, which can be interpreted as close inspection/inactivity within a narrow region of the test surface rather than swimming.

**Fig. 6:**
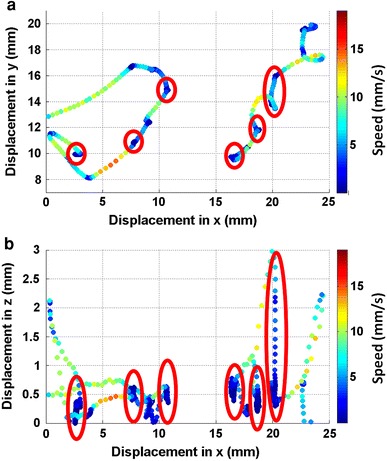
Two trajectories of*Semibalanus balanoides* cyprids, exploring a glass surface in *xy* representation (**a**) and *xz* representation (**b**). In the *red circled* regions of subplot (**a**) it seems like a close inspection of the surface is taking place at low speeds, but if the third dimension is considered (**b**) it becomes obvious that these regions represent gravity induced passive sinking phases

In Fig. 6a, the red marked regions look like regions of close inspection on the surface with a relatively low speed. But if we look at Fig. 6b we can see that even though there is no spatial displacement in the X and Y direction, the displacement in Z is significant (a slow motion towards the surface). This phenomenon might be seen as passive, gravity-induced sinking of the cyprids and is only visible if three dimensional data is available.

**Fig. 7:**
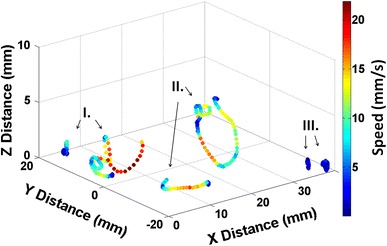
Swimming trajectories of six cyprids of the species*Semibalanus balanoides*, exploring three different surfaces: glass (*I*), PEG (*II*) and TMA (*III*). Velocities are encoded by the*color*

In order to provoke surface specific exploratory behavior, a test with surfaces leading to a different settlement behavior was performed. The work done by Holmlin et al. [26] and Ekblad et al. [27] showed that the surface charge plays an important role in the adsorption of proteins on surfaces and that surfaces with positive and negative charges have an attractive effect while surfaces with neutral charge, such as zwitterionic surfaces, are rather inert. We selected a positively charged surface (TMA) and compared cyprid behavior to polyethylene glycol (PEG) surfaces in our first tests. PEG surfaces have been reported to be effectively resistant against a range of biofouling organisms including barnacle cyprids [28]. Figure 7 shows the trajectories of cyprids exposed to the selected test surfaces (TMA, PEG and glass as a standard surface commonly employed in settlement assays). For each of the surfaces, two representative traces are shown and all six traces are combined into a single figure. The glass traces (Fig. 7-I.) are included just for qualitative comparison.

In the region far away from the surface (2–10 mm), the traces for PEG (Fig. 7-II.) are qualitatively similar compared to the traces for glass. In agreement with the above discussion, trajectories extending into solution reveal in general relatively high swimming velocities (up to 10–25 mm/s) combined with a wide exploration region. On TMA, no volume trajectories were found as cyprids mostly stayed close to the surface (Fig. 7-III.). The analysis of motility close to the surface reveals that in the case of PEG the swimming velocities are still in the order of 10–20 mm/s and do not change significantly compared to motility further away from the surface. Also, the perimeter of exploration remains relatively wide. On the TMA surfaces, all the movements are highly localized at very low velocities (<5 mm/s). The cyprids stay on the surface during the whole observation period and seem to explore it slowly and thoroughly, while turning around in circles in a relatively narrow exploration region.

**Fig. 8:**
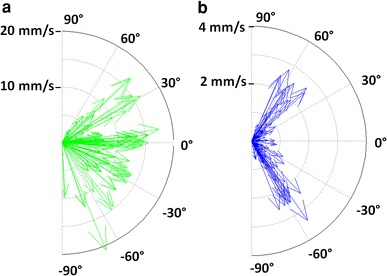
Polar combination plot showing the swimming speed (vector length) versus swimming angle (angle between the cyprid and the surface) of cyprids over (**a**) PEG and (**b**) TMA surfaces. Note the different scaling of the r-axis (swimming speeds) which is in general much larger on PEG

To quantify these descriptive observations, the swimming angle, with respect to a vector in the surface plane, was analyzed in relation to the cyprid velocity (Fig. 8). In general, the velocities on the TMA surfaces were much lower compared to PEG (note the different velocity scales in the polar plots). In addition to the velocity difference, larger angles were more frequently observed close to the TMA surface and motions parallel to the surface were mostly very slow. Larger velocities were observed when motions towards or away from the surface occurred under an angle of ≈60° with respect to the surface plane. On PEG surfaces, the motion seems much less affected by the surface and even in the vicinity both vector distribution and angular-depending-velocity-change seem isotropic.

**Fig. 9:**
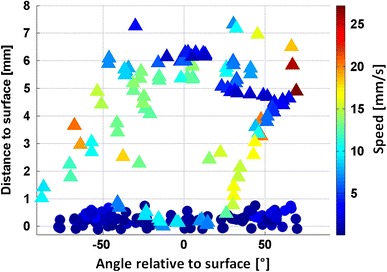
Angle versus distance to the surface graph, including the swimming velocity (coded in*color*). The PEG values (*triangles*) expose generally higher velocities when compared to the TMA values (*circles*)

This analysis is further supported by Fig. 9, which shows a combined plot of the distribution of the swimming speed (color) and the distance to the surface (y axis) over the angle relative to the surface (x axis) for PEG (triangles) and TMA (circles) surfaces. Again it is important that speeds are much higher in the case of PEG and that indicates that cyprids are moving much faster and are less decelerated by the PEG surfaces (values between 10 and 20 mm/s in close proximity and on the surface). In the case of exploration of the TMA surfaces (Fig. 9, circles), the overall velocities are much slower and almost exclusively on the surface, which can be interpreted as stronger interaction with the positively charged surfaces.

As mentioned above, the influence of surface charge on the resistant properties of surfaces has been studied extensively in the past. However, the influence of the presence of tethered, positively charged ammonium groups on barnacle cyprids is still equivocal. Petrone et al. [29] found higher settlement (50 %) of cyprids of the barnacle *B. amphitrite* on negatively charged (carboxyl) and only low settlement (<5 %) for positively charged surfaces (quaternary amines). Using cyprids of *Semibalanus balanoides*—the same species as in this study—deposition of the proteinaceous temporary adhesive (‘footprints’) has been investigated by Aldred et al. [2] using iSPR. It was found that the number of touchdowns made by the cyprids on chemically different surfaces did not differ significantly and was independent of surface charge. Also, the probability of leaving adhesive ‘footprints’ during exploration was ≈20 % on both, positively charged ammonium terminated surfaces (–NH_3_^+^) and on ethylene glycol terminated surfaces (mPEG). However the amount of deposited footprint material was found to be significantly higher on the positively charged surfaces compared to the mPEG coating. The fact that in both cases (–NH_3_^+^ and mPEG) only ≈20 % of the touchdowns resulted in footprints, but the amount of deposited material was quite different, suggests that footprint deposition could be connected with the resistant properties of the surfaces. Our observation that extensive exploration is observed on TMA is a further evidence for attractive cues present on these surfaces.

It is important to note that in this work we only attempted quantification of a limited number of swimming trajectories and described observations that were made to demonstrate the applicability of our stereoscopic technique to record surface exploration behavior. For future work a more sophisticated full statistical analysis will allow a quantitative correlation of surface properties and behavior. However, the results shown here already demonstrate that stereoscopy allows investigating how barnacle cyprids select the suitability of a surface for settlement.

## Summary and Conclusions

A stereoscopic tracking system for sub-millimeter sized microorganisms has been developed and the theoretical framework for its calibration and use has been described. The physical resolution of the cameras, the 3D resolution in position determination, and the frame rate are suited to track the exploration behavior of the target larvae. As shown in the analysis section, the errors originate mainly from position uncertainties in both the calibration and the tracking steps, and accumulate to less than 1 % of the size of the larvae. Thus, the precision is sufficient for very accurate 3D tracking.

The future key application will be to track larvae of biofouling organisms [30]. Barnacle cyprids are of primary interest as they are widely distributed and have been identified as the most common fouling marine invertebrates in the world [31, 32]. The first results obtained with our stereoscopic setup and cyprids of the barnacle *Semibalanus balanoides* demonstrated successful tracking and showed that differences in the swimming trajectories in solution and close to surfaces could be detected. First indications were obtained which suggested that the surface properties affected the exploration behavior at the surface. Even though all results presented need to be statistically verified with more comprehensive data sets, they clearly show the applicability of the experimental approach. As the setup is very compact and highly modular, a future implementation and tests in situ in the ocean will allow investigating the colonization of surfaces by biofouling larvae in their natural habitat. The developed device can easily be applied for the 3D analysis not only of organisms at sub-millimeter level but also for larger animals, and thus has numerous potential applications in biology, biochemistry, and biophysics.

One question arising with increasing quantities of data is the automatic analysis which can be realized as demonstrated for smaller organisms by Leal-Taixé et al. [8] or even using more powerful 3D tracking techniques specifically designed for stereoscopic systems [14]. The same holds true for the subsequent automated classification of motion patterns, which is required to show that changes in the occurrence of patterns as result of surface cues are reproducible and have statistical relevance [20]. We see great potential to even combine 3D tracking with further emerging techniques which are sensitive towards interaction of the cyprid temporary adhesive with surfaces, such as imaging SPR as recently reported by Andersson et al. [33].

The developed 3D tracking system will allow to gain a deeper understanding of the different stages of attachment of larvae of barnacles and other biofouling organisms to interfaces. Besides the relevance of such information for understanding behavior, responses to physical and chemical cues and sensory capabilities of the larvae, comparisons of different surfaces will support the development of environmentally friendly antifouling concepts, interfering at crucial stages of the surface selection process.

## Electronic supplementary material

Below is the link to the electronic supplementary material.

Supplementary material 1 (PDF 515 kb)
