# Supplementary material for: Three Dimensional Tracking of Exploratory Behavior of Barnacle Cyprids Using Stereoscopy
Source: Biointerphases. 2012 Aug 21;7(1-4):50. doi: 10.1007/s13758-012-0050-x (PMC4875145; doi:10.1007/s13758-012-0050-x)
Supplement: Supplementary file 1 — Supplementary material 1 (PDF 515 kb) [file 13758_2012_50_MOESM1_ESM.pdf]

Supplemental Information

Three dimensional tracking of exploratory behavior of barnacle cyprids using stereoscopy

S. Maleschlijski[1], G. H. Sendra[1,2], A. Di Fino[3], L. Leal-Taixé[4], I. Thome[1], A. Terfort[5], N. Aldred[3], M. Grunze[1,2], A. S. Clare[3], B. Rosenhahn[4], A. Rosenhahn[1,2]

[1]*Institute of Functional Interfaces, KIT, PO Box 3640, 76021 Karlsruhe, Germany*

[2]*Applied Physical Chemistry, University of Heidelberg, INF 253, 69120 Heidelberg, Germany*

[3]*School of Marine Science and Technology, Newcastle University, Newcastle upon Tyne NE1 7RU, UK*

[4]*Institute for Information Processing, Leibniz University Hannover, Appelstr. 9A, Hannover, Germany*

[5]*Institute of Inorganic and Analytical Chemistry, Goethe University Frankfurt, D-60438 Frankfurt, Germany*

Corresponding author:

Stojan Maleschlijski

Tel: +49721608-28227

Email: stojan.maleschlijski@kit.edu

S.1. Noise analysis of the accuracy in the position determination

The calculation of the projection matrices during system calibration introduces an error due to the finite precision of the floating point calculations in the software (rounding error). While systematic errors can be overcome using some caution during calibration, noise induced by morphing of non-spherical objects and dirty water can also affect the measurements. For the analysis of a displacement of the detected cyprid position in real life coordinates, a random offset signal (with special characteristics) is added to the coordinates of the cyprid in the left and right frame, thus simulating a random error in the position determination.

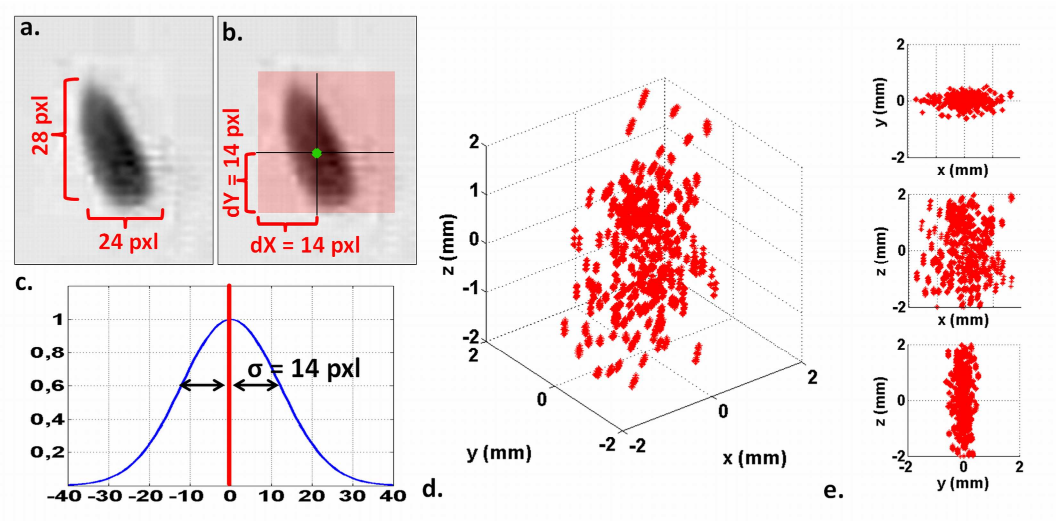

Fig. 1: 3D position noise analysis. Considering the mean size of a cyprid (a) the maximal realistic error (deltaX and deltaY) was calculated (b) (this error would result in a completely wrong centroid estimation). Assuming that the error in the cyprid position determination in both left and right frame has Gaussian distributed probability function with the values estimated in (b), an error signal was generated and added to the calculation values of the algorithm (c). Adding this noise, creates an “error cloud” in the real position of the cyprid in three dimensional space (d, e) (The value (0,0,0) represents the correct position).

The parameters of the noise signal are determined based on the maximal relevant and possible theoretical error. As the average size of the cyprids in the images is around 28 x 24 pixels (**Fig. 1**a), the maximal realistic error is assumed to be 14 pixels in both x and y directions. This error means that the detection algorithm estimate the coordinates of a cyprid with a displacement equivalent to its size (**Fig. 1**b), which would correspond to a 100% error. If we assume that the probability of occurrence of an error in the calculation has a Gaussian distribution, we can say that the standard deviation of this error probability will be 14 pixels

(**Fig. 1**c) in the worst case (meaning that errors in the position determination in the range of -14 to 14 pixel will have the highest probability to occur).

To test the approach, the error in position determination of 24 representative points at different positions on the calibration object was investigated. It is important to include points from different positions, because the error values of the system are position dependent and need to be analyzed together in order to get a pseudo-mean value of the error of the whole setup. The procedure of adding the Gaussian zero mean noise to the coordinates is repeated for all of the test points. The difference between the real 3D position of the test point and its calculated position with noisy input is accumulated and the result is shown in **Fig. 1** (d and e). The effect of noise on the 3D position is extremely low in the y-direction compared to the *x* and *z* directions. The low *y* values are a consequence of the geometry of the setup, since both cameras “look” at the calibration object from two different *x* and *z* positions, but from nearly the same *y* position. This means that they share the same virtual *y*-axis (**Fig. 1**, in article) in their respective coordinate systems, which minimizes the triangulation error and simplifies the projection and fundamental matrix calculations. If we consider a full error in position determination (error values of 14 pixels in both, x and y directions of the left and right recorded frames), shifts of 1.7 mm (170% of the cyprid size in mm) are the consequence in the 3D position. However in reality the center of mass of the bright spots can be determined with an accuracy of at least 2 pixels. With an offset of 2 pixels (8% of cyprid size in pixel) in x and y, the shift results to be 0.08 mm (8% of cyprid size in mm).

S.2. Systematic Characterization of the accuracy in the position determination

In order to prove the random noise approach and investigate the dependency of the system on the geometrical setup a systematic characterization test is needed. However, the relationship between the error in the real position (in mm) and in the position of the object on the left and the right frames (in pixel) needs to be determined empirically. For this purpose the error in the position of a test object in one of the frames was gradually increased in *x* and *y*, while its position in the other frame was kept constant (**Fig. 2**). The *x* and *y* axes represent the error in pixels of the position of the test object in the left frames (a) and the

right frame (b). A deviation of up to 14 pixels was investigated, which corresponds to the radius of the cyprid (see **Fig. 2**b, article). The *z* axis of **Fig. 2** shows the magnitude of the resulting 3D error in mm. This error is decomposed in its three components *X*, *Y* and *Z*, each of them appearing as a separate plane in the diagram.

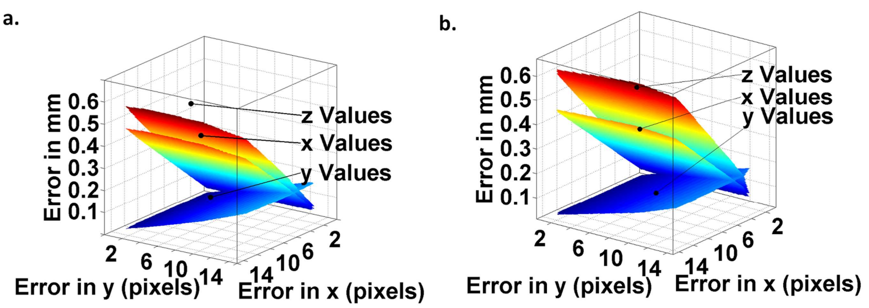

Fig. 2: Relationship between pixel errors in the object coordinates of the image frames and the resulting error in real life coordinates of the object in mm, when an error is introduced in the left (a) or right (b) frame. The three planes resemble the 3D position error in the X, Y and Z position, respectively.

Fig. 2 shows that the error components in the ***X*** and ***Z*** direction are much higher than the one in the ***Y*** direction. This can be explained by the fact that the position determination is done via triangulation of the back-projected rays starting from the position pairs in the left and the right frame. Since these rays do not necessarily intersect, the crossing point is assumed as the closest point to both of them. On the other hand, both cameras share the same virtual *y*-axis, hence increasing the *x* error in pixels does not cause a considerable change in the *Y* error in mm, which is mainly affected by the error in the *y* direction.

It is important to mention that the maximal error in the position of the cyprid in the image frames (14 pixels ≈ 100% error in the cyprid size), investigated in this experiment, introduced an error in the position in real coordinates of 0.5 mm (≈ 50% of physical cyprid size) in *X* and *Z*, and 0.2 mm (≈ 20% of physical cyprid size) in *Y*. These are worst-case estimations, because an error of 14 pixels in the position determination would mean that the centroid would lie completely outside of the cyprid body (see **Fig. 1**b). Based on our experience and observations, an error of max 2 pixels is typical for this application. This means that the typical error of the system related to the size of the cyprids is around 0.08 mm (~8% of physical size of *Semibalanus balanoides* cyprids), which is in the same range as the error determined from the noise analysis as described above.

There is a linear relationship between the error in pixels and the error in mm which is a logical consequence of the linear operations during the stereoscopic calculations. There is still some uncertainty in the form of the error function for points located near the edges of the camera, where possible optical distortions of higher orders could have a greater impact.
